# Detecting retinal neurodegeneration in people with diabetes: Findings from the UK Biobank

**DOI:** 10.1371/journal.pone.0257836

**Published:** 2021-09-29

**Authors:** Roomasa Channa, Kyungmoo Lee, Kristen A. Staggers, Nitish Mehta, Sidra Zafar, Jie Gao, Benjamin J. Frankfort, Sharon Y. L. Chua, Anthony P. Khawaja, Paul J. Foster, Praveen J. Patel, Charles G. Minard, Chris Amos, Michael D. Abramoff

**Affiliations:** 1 Department of Ophthalmology and Visual Sciences, University of Wisconsin, Madison, WI, United States of America; 2 Department of Ophthalmology and Visual Sciences, University of Iowa, Iowa City, Iowa, United States of America; 3 Institute for Clinical and Translational Research, Baylor College of Medicine, Houston, TX, United States of America; 4 New York University, New York, NY, United States of America; 5 Wilmer Eye Institute, Johns Hopkins University, Baltimore, MD, United States of America; 6 Department of Ophthalmology, Baylor College of Medicine, Houston, TX, United States of America; 7 Department of Ophthalmology and Neurosciences, Baylor College of Medicine, Houston, TX, United States of America; 8 NIHR Biomedical Research Centre at Moorfields Eye Hospital NHS Foundation Trust & UCL Institute of Ophthalmology, London, United Kingdom; Massachusetts Eye & Ear Infirmary, Harvard Medical School, UNITED STATES

## Abstract

**Importance:**

Efforts are underway to incorporate retinal neurodegeneration in the diabetic retinopathy severity scale. However, there is no established measure to quantify diabetic retinal neurodegeneration (DRN).

**Objective:**

We compared total retinal, macular retinal nerve fiber layer (mRNFL) and ganglion cell-inner plexiform layer (GC-IPL) thickness among participants with and without diabetes (DM) in a population-based cohort.

**Design/setting/participants:**

Cross-sectional analysis, using the UK Biobank data resource. Separate general linear mixed models (GLMM) were created using DM and glycated hemoglobin as predictor variables for retinal thickness. Sub-analyses included comparing thickness measurements for patients with no/mild diabetic retinopathy (DR) and evaluating factors associated with retinal thickness in participants with and without diabetes. Factors found to be significantly associated with DM or thickness were included in a multiple GLMM.

**Exposure:**

Diagnosis of DM was determined via self-report of diagnosis, medication use, DM-related complications or glycated hemoglobin level of ≥ 6.5%.

**Main outcomes and measures:**

Total retinal, mRNFL and GC-IPL thickness.

**Results:**

74,422 participants (69,985 with no DM; 4,437 with DM) were included. Median age was 59 years, 46% were men and 92% were white. Participants with DM had lower total retinal thickness (-4.57 μm, 95% CI: -5.00, -4.14; p<0.001), GC-IPL thickness (-1.73 μm, 95% CI: -1.86, -1.59; p<0.001) and mRNFL thickness (-0.68 μm, 95% CI: -0.81, -0.54; p<0.001) compared to those without DM. After adjusting for co-variates, in the GLMM, total retinal thickness was 1.99 um lower (95% CI: -2.47, -1.50; p<0.001) and GC-IPL was 1.02 μm lower (95% CI: -1.18, -0.87; p<0.001) among those with DM compared to without. mRNFL was no longer significantly different (p = 0.369). GC-IPL remained significantly lower, after adjusting for co-variates, among those with DM compared to those without DM when including only participants with no/mild DR (-0.80 μm, 95% CI: -0.98, -0.62; p<0.001). Total retinal thickness decreased 0.40 μm (95% CI: -0.61, -0.20; p<0.001), mRNFL thickness increased 0.20 μm (95% CI: 0.14, 0.27; p<0.001) and GC-IPL decreased 0.26 μm (95% CI: -0.33, -0.20; p<0.001) per unit increase in A1c after adjusting for co-variates. Among participants with diabetes, age, DR grade, ethnicity, body mass index, glaucoma, spherical equivalent, and visual acuity were significantly associated with GC-IPL thickness.

**Conclusion:**

GC-IPL was thinner among participants with DM, compared to without DM. This difference persisted after adjusting for confounding variables and when considering only those with no/mild DR. This confirms that GC-IPL thinning occurs early in DM and can serve as a useful marker of DRN.

## Introduction

Globally there are an estimated 93 million people with diabetic retinopathy (DR) and 21 million with diabetic macular edema (DME) [1]. Vision loss from diabetic retinal diseases is thought to be primarily due to vascular damage, which manifests as DR or DME. However, there is increasing evidence of an underlying neurodegenerative process [2, 3] and recognition regarding the need to update the DR severity scale to incorporate diabetic retinal neurodegeneration(DRN) [4, 5]. Spectral Domain Optical Coherence Tomography (SD-OCT) is the most widely available imaging technique to quantify structural changes in the neuroretina. While prospective studies have shown that DRN occurs before DR manifests [6], results from cross-sectional studies evaluating retinal thickness parameters to quantify DRN have reported mixed results, have included small samples and have not always controlled for the many factors that can impact thickness measurements including, age, gender, ethnicity, refractive error, cognitive impairment, glaucoma, systemic co-morbidities and medications [7–10]. Consequently, some studies report increased thickness of inner retinal layers, some show no changes and others suggest that the RNFL and GC-IPL may be thinner in patients with DM compared to controls [11, 12]. Lim et al reported a higher rate of peripapillary RNFL thinning among patients with DM compared to those without DM [13]. Although this information is useful, clinically it is helpful to identify changes in the macula as macula-centered OCT scans are already routinely used to evaluate macular edema in patients with diabetes. A recent study of 112 patients with diabetes and 63 healthy controls reported a higher rate of GC-IPL thinning among patients with diabetes, but did not report changes in the macular RNFL [14]. Also, given the small number of patients in this study it is difficult to fully evaluate factors associated with inner retinal thickness changes in patients with diabetes. If we are to incorporate evaluation of DRN in clinical practice, using OCT scans, it is important to fully understand factors associated with inner retinal thickness measurements in participants with and without diabetes. Data from population-based studies such as the UK Biobank cohort, offer the opportunity to evaluate changes in RNFL and GC-IPL thickness in a large number of participants with and without diabetes. Additionally, these data allow us to evaluate the impact of systemic and ocular conditions on retinal thickness measurements providing important information regarding factors to consider when using OCT-based thickness changes in the evaluation of DRN.

We aimed to identify structural changes to the neuroretina from diabetes, by comparing total retinal thickness and retinal thickness of the neural unit i.e., soma + dendrite, identified as ganglion cell-inner plexiform layer (GC-IPL) and axon as identified by retinal nerve fiber layer (RNFL) on macula centered SD-OCT scans of participants with and without DM in a large population-based cohort. We also evaluated the impact of A1c level on retinal thickness measurements, and factors associated with retinal thickness measurements in participants with diabetes.

## Methods

### Study population

The UK Biobank is a large population-based cohort of over 500,000 participants [15]. Detailed study protocols are available online (http://www.ukbiobank.ac.uk/resources/ and http://biobank.ctsu.ox.ac.uk/crystal/docs.cgi). Briefly, from 2006–10, all residents of the United Kingdom, between the ages of 40 to 69 years, who were registered with the National Health Service were invited to participate. The study was conducted in accordance with the principles of the declaration of Helsinki and approved by the ethics committee and institutional regulatory boards, and all participants gave informed consent. Participants initially completed a baseline questionnaire related to demographics, medical history, medication use, as well as lifestyle. This was followed by a verbal interview by a nurse, physical exam, blood draw and additional measures [15]. Ophthalmic assessment was added in 2009 for selected assessment centers. This included measurement of visual acuity, intraocular pressure, refractive error, fundus photos and SD-OCT scans. All participants who had SD-OCT measures were considered for inclusion in the study.

### Retinal imaging in the UK Biobank

Macular SD-OCT scans plus a one-field 45-degree fundus photograph were acquired using a Topcon camera (Topcon 3D OCT-1000 Mark II; Topcon GB). The SD-OCT image acquisition protocol is described in detail in a previous publication [16]. Participants who had an eye infection or any eye surgery within the last 4 weeks did not undergo any ocular measurements. For this study we used Iowa Reference Algorithm software version 3.8 to determine 1) total retinal thickness 2) the thickness of the mRNFL and 3) GC-IPL. This software has been previously demonstrated to give accurate and reliable thickness measurements [17–19]. Original image files were downloaded from the UKBB servers. Two automated measures of image quality were used: 1) undefined region and 2) surface cost. Undefined region quantified the percentage area of a scan that had missing data or insufficient signal. Images with undefined region > 0% were excluded from analysis. The surface cost is calculated using edge-based costs of the dark to bright and bright to dark transition of the retinal sub-layers. This is an inverted Gaussian-smoothed gradient magnitude of the OCT voxel intensities of the retinal sub-layers. A lower value corresponds to more reliable segmentation [20]. We randomly evaluated 100 OCT scans with a range of surface cost values and determined that scans with surface cost values > 62, 000 had unreliable segmentation. Subsequently all scans with surface cost values > 62, 000 were excluded. For the remaining OCT images, any scans with thickness measurements 2 or more standard deviation above or below the mean were manually reviewed. Scans with incorrect algorithm, poor quality, poor signal or presence of pathology such as epiretinal membrane, macular edema, drusen, intra-retinal or sub-retinal fluid, pigment epithelial detachment, decentration that could impact thickness measurements were excluded.

A previously validated procedure was used to determine DM status based on self-reported DM diagnosis, use of DM medications and presence of DM complications [21]. We also used HbA1c ≥ 6.5% as a criterion for identifying DM. Two graders (NM and JG) independently evaluated the fundus photos for quality and graded DR based on the International Clinical Diabetic Retinopathy Scale as no/mild DR (no changes or microaneurysms only) or more than mild DR [22]. Final DR grade was determined by the higher of the two grades. We defined glaucoma as self-reported diagnosis of glaucoma, taking glaucoma medications, or having had glaucoma surgery. We defined cognitive impairment as a failing score on 2 or more tests as has been described in prior publication [23].

### Exclusions

Participants were excluded if they withdrew consent, were identified to have pre-diabetes or gestational diabetes; if they had poor quality OCT scans as described above, eyes affected by injury or trauma resulting in loss of vision; eyes affected by retinal pathology as described above which could distort the retinal layers; those with self-reported history of retinal surgery or macular degeneration; extreme IOP values of less than 5mmHg or more than 60mmHg.

### Statistical analysis

Patient and eye characteristics were summarized using mean with standard deviation, median with minimum and maximum values, or frequency with percentage. Summary statistics were stratified by diabetes. Patient summary statistics were compared using the Wilcoxon rank sum, Fisher’s exact or Chi-square test. Eye summary statistics were compared using generalized estimating equations (GEE) with a logit link and exchangeable correlation. Independent general linear mixed models (GLMM) with a random intercept for patient and eye were used to test the association between patient/eye characteristics and SD-OCT thickness measurements (RNFL, GC-IPL, and total retinal thickness). Factors found to be significantly associated with DM or thickness, or those that were clinically significant were included in a multiple GLMM. When correlation between predictor variables was > 0.5, only one was included in the GLMM to avoid multicollinearity. Separate GLMMs were created using DM and A1c as predictor variables for thickness. Sub-analyses were done 1) including only patients with no/mild DR in the DM group, and 2) to evaluate factors associated with GC-IPL thickness specifically for DM patients. In addition to the co-variates used in other models, the analysis with only DM patients tested whether thickness differed for patients who had an A1c > = 6.5% or who were on metformin, whether they had DR and the duration of DM. P < 0.05 was considered statistically significant.

## Results

74,422 participants (131,555 eyes) met all inclusion and exclusion criteria described above and were included in the analysis. 10,024 participants (35,096 eyes) were excluded from the analysis after quality control and other exclusion criteria described above. Comparison of summary statistics between those included and excluded in the analyses are presented in S1A and S1B Tables and a detailed list of the number of patients/eyes excluded from analysis is shown in S2 Table.

Of the 74,422 participants included in the study: 69,985 did not have DM and 4,437 had DM. Participants with DM were older (median 62.3 years vs 59 years, p<0.001), more likely to be men (63.0% vs 45.2%, p<0.001), more likely to be on cholesterol lowering (45.1% vs 9.5%, p<0.001) or blood pressure lowering medications (39.2% vs 9.9%, p<0.001), and more likely to have impairment on cognitive testing (29.6% vs 19.3%, p<0.001) compared to those without DM (See Tables 1and 2). Participants with DM had on average lower total retinal thickness (4.57 μm lower, 95% CI: -5.00, -4.14; p<0.001), GC-IPL thickness (1.73 μm lower, 95% CI: -1.86, -1.59; p<0.001) and mRNFL thickness (0.68 μm lower, 95% CI: -0.81, -0.54; p<0.001) compared to those without DM. A full list of the associations with total retinal thickness, mRNFL and GC-IPL thickness measures are shown in S3 Table. Systolic blood pressure and diastolic blood pressure were correlated and use of cholesterol lowering and blood-pressure lowering medications were correlated. To avoid collinearity, we chose systolic blood pressure and cholesterol lowering medication for the multivariable GLMM.

**Table 1:** Patient summary statistics stratified by diabetes (all included patients).

Characteristics	N	No diabetes (N = 69,985)	N	Diabetes (N = 4,437)	p-value
**Age(yrs), median (min, max)**	69,985	59 (39.2,76.5)	4,437	62.3(40.2,75.9)	<0.001
**Education scores median (min, max)**	68,581	8.7 (0.0,96.2)	4,339	12.2 (0.0,93.0)	<0.001
**BMI, median (min, max)**	69,669	26.4 (12.6,66.0)	4,403	30.2 (17.7,60.4)	<0.001
**HDL, median (min, max)**	60,207	1.5 (0.4,4.1)	3,796	1.2 (0.4,3.8)	<0.001
**Diastolic BP, median (min, max)**	69,749	81 (39.5,142.0)	4,419	81(44.5,122.5)	0.403
**Systolic BP, median (min, max)**	69,748	135.5 (65.0,241.0)	4,419	139.5 (84.5,212.0)	<0.001
**Female, n (%)**	69,985	38,363(54.8)	4,437	1,643 (37.0)	<0.001
**Ethnicity white, n (%)**	69,558	64,321 (92.5)	4,389	3,592 (81.8)	<0.001
**Incorrect answer on first attempt of prospective memory, n (%)**	69,628	14,337 (20.6)	4,385	1,313 (29.9)	<0.001
**>2 incorrect matches on pairs matching, n (%)**	69,849	44,472 (63.7)	4,421	2,957 (66.9)	<0.001
**Fluid intelligence score < 3, n (%)**	67,863	2,670 (3.9)	4,120	290 (7.0)	<0.001
**Reaction time > 770 milliseconds, n (%)**	69,250	3,735 (5.4)	4,348	392 (9.0)	<0.001
**Cognitive impairment, n (%)**	68,954	13,331 (19.3)	4,298	1,272 (29.6)	<0.001
**BP medication, n (%)**	69,985	6,949 (9.9)	4,437	1,740 (39.2)	<0.001
**Cholesterol lowering medication, n (%)**	69,985	6,683 (9.5)	4,437	2,003 (45.1)	<0.001

p-values for median comparisons using two-sample Wilcoxon rank-sum (Mann-Whitney) test; p-values calculated with exact testing for categorical variables when possible otherwise chi-square test; BMI = body mass index, HDL = high density lipoprotein, BP = blood pressure.

**Table 2:** Eye summary statistics stratified by diabetes (all included eyes).

Variable	N	No diabetes (N = 123,868	N	Diabetes (N = 7,687)	p-value
**logMAR, median (min, max)**	123,674	-0.04 (1.04,1.35)	7,662	0.00 (-1.06,1.35)	0.020
**Spherical equivalent (diopters), median (min, max)**	122,636	0.2 (-21.1,14.0)	7,578	0.2 (-19.8,10.1)	0.083
**IOP (mmHg) median (min, max)**	120,535	15.4 (5.0,59.8)	7,473	16 (5.5,51.3)	0.001
**Cataract surgery, n. (%)**	123,863	1,538 (1.2)	7,687	268 (3.5)	<0.001
**Glaucoma, n. (%)**	123,868	1,560 (1.3)	7,687	164 (2.1)	0.077
**Total retinal thickness (μm), mean (SD)**	123,868	310.6 (14.1)	7,687	306.1 (15.2)	<0.001
**Retinal nerve fiber layer thickness (μm), mean (SD)**	123,868	30.1 (3.4)	7,687	29.5 (3.5)	<0.001
**Ganglion Cell-Inner Plexiform layer (μm), mean (SD)**	123,868	70.2 (6.2)	7,687	68.5 (6.5)	<0.001

p-values estimated using independent GEE; IOP = intraocular pressure.

**Fig 1:**
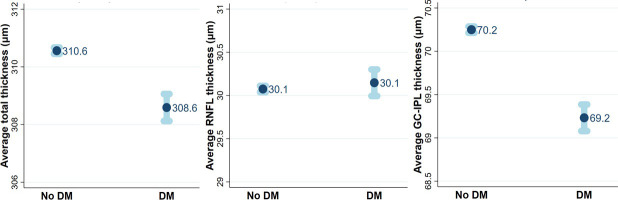
Difference in retinal thickness measurements between participants with and without diabetes. Fig 1 shows the difference in average thickness measurements (95% confidence interval) between participants with and without diabetes (DM) after adjusting for co-variates: a) total retinal thickness, b) retinal nerve fiber layer (RNFL) thickness, c) ganglion cell inner plexiform layer (GC-IPL) thickness.

**Fig 2:**
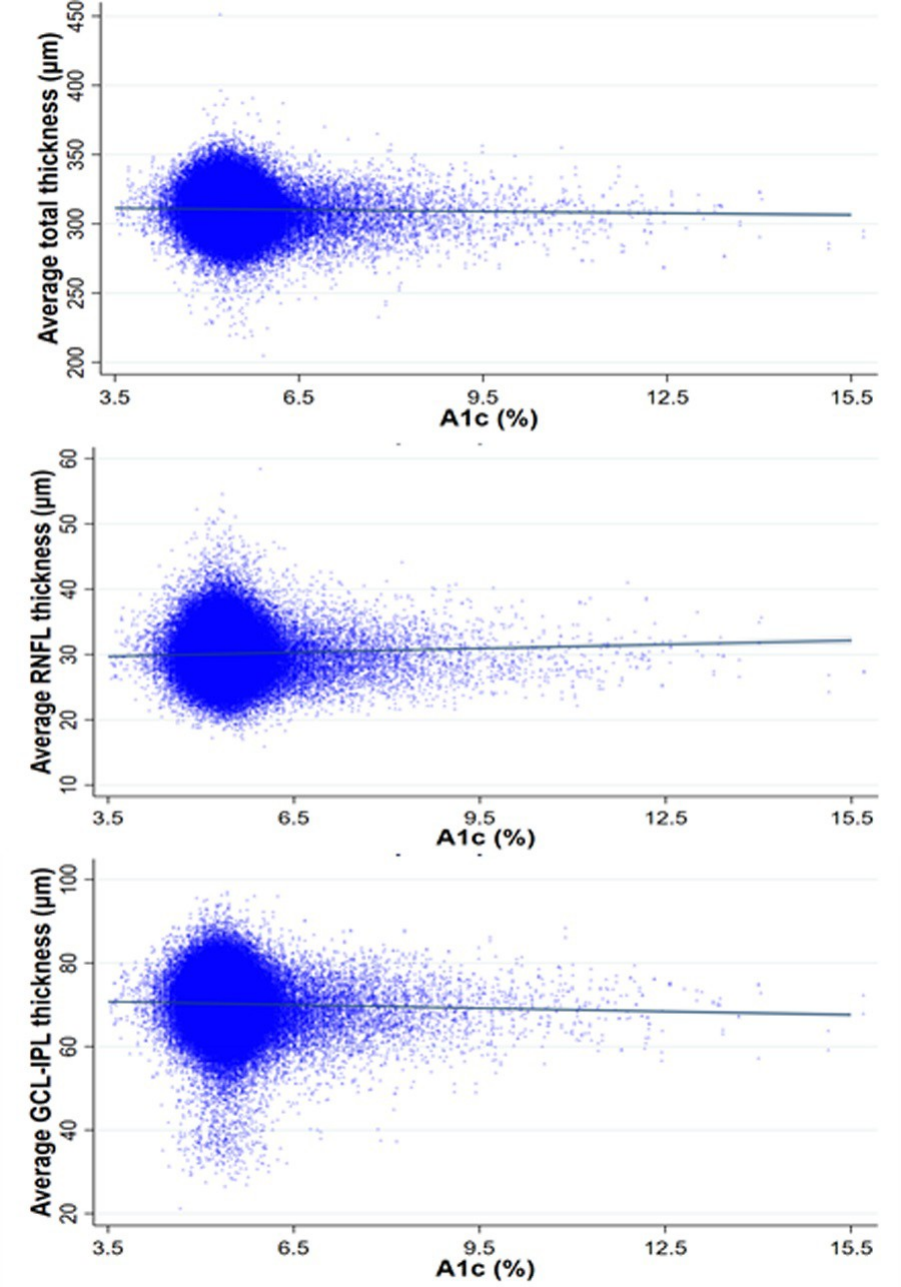
Change in retinal thickness measurements with change in glycated hemoglobin levels. Fig 2 shows the change in a) average total retinal thickness, b) average retinal nerve fiber layer (RNFL) thickness, c) average ganglion cell-inner plexiform (GC-IPL) layer thickness after adjusting for co-variates.

After adjusting for co-variates in the multivariable GLMM (See Table 3), total retinal thickness was 1.99 um thinner (95% CI: -2.47–1.50, p<0.001) among participants with diabetes compared to those without DM, and GC-IPL was 1.02 um lower (95% CI: -1.18, -0.87; p<0.001) among those with diabetes compared to those without. The difference in mRNFL thickness was no longer significantly different (p = 0.369). Fig 1A–1C show the adjusted differences in average retinal thickness measurements after controlling for co-variates among participants with and without DM for total retinal thickness (Fig 1A), mRNFL (Fig 1B) and GC-IPL thickness (Fig 1C). On average, total retinal thickness decreased 0.40 um (95% CI:-0.61, -0.20; p<0.001), mRNFL thickness increased 0.20 um (95% CI: 0.14, 0.27; p<0.001) and GC-IPL decreased 0.26 um (95% CI: -0.33, -0.20; p<0.001) per unit increase in A1c, after adjusting for co-variates. See Fig 2A–2C.

**Table 3:** Multivariable general linear mixed models for retinal thickness measurements.

	Total retinal thickness				RNFL/GC-IPL thickness			
	Coefficient	95% Confidence Interval		p-value	Coefficient	95% Confidence Interval		p-value
**Location**								<0.001
RNFL					Reference	.	.	.
GCL-IPL					40.17	40.14	40.21	<0.001
**DM**	-1.99	-2.47	-1.50	<0.001	0.07	-0.09	0.23	0.369
GCL-IPL * DM					-1.10	-1.23	-0.97	<0.001
**Age (10 year increase)**	-3.34	-3.49	-3.19	<0.001	-0.90	-0.94	-0.85	<0.001
**Male**	4.68	4.43	4.93	<0.001	0.23	0.15	0.30	<0.001
**Ethnicity (Other than White)**	-6.64	-7.06	-6.23	<0.001	-1.17	-1.30	-1.05	<0.001
**Cognitive impairment**	-0.46	-0.73	-0.18	0.001	-0.10	-0.18	-0.02	0.015
**Education score**	-0.02	-0.03	-0.01	<0.001	-0.01	-0.01	-0.01	<0.001
**BMI**	-0.13	-0.16	-0.11	<0.001	-0.02	-0.03	-0.02	<0.001
**Systolic BP**	-0.01	-0.02	0.00	0.003	-0.01	-0.01	0.00	<0.001
**HDL**	1.06	0.74	1.38	<0.001	-0.12	-0.22	-0.03	0.013
**Cholesterol lowering medication**	-1.92	-2.30	-1.54	<0.001	-0.49	-0.60	-0.38	<0.001
**Glaucoma**	-5.72	-6.33	-5.12	<0.001	-3.33	-3.58	-3.08	<0.001
**Cataract surgery**	3.36	2.80	3.92	<0.001	0.55	0.31	0.79	<0.001
**Spherical equivalent (diopters)**	1.14	1.10	1.17	<0.001	0.13	0.12	0.14	<0.001
**logMAR**	-2.20	-2.42	-1.98	<0.001	-1.22	-1.34	-1.09	<0.001
**IOP (mmHg)**	-0.03	-0.04	-0.01	<0.001	-0.02	-0.02	-0.01	<0.001

BMI = body mass index, HDL = high density lipoprotein, BP = blood pressure, iop = intraocular pressure, RNFL = retinal nerve fiber layer, GC-IPL = ganglion cell inner plexiform layer.

### Factors associated with GC-IPL thickness among participants with DM

We further evaluated the factors associated with GC-IPL thickness specifically among participants with DM. Average GC-IPL thickness was not significantly different between patients who did and did not have A1c> = 6.5% (p = 0.120), and was not associated with duration of DM (p = 0.172). Average GC-IPL was 1.24 um thinner among those with more than mild DR compared to those with no/mild DR (95% CI: -1.89, -0.59; p<0.001). See Table 4 for multivariable model of the factors associated with GC-IPL thickness among participants with DM. This sub-analysis included 1,959 participants (3,239 eyes) with no/mild DR and 146 participants (190 eyes) with more than mild DR.

**Table 4:** Multivariable general linear mixed model for GC-IPL thickness among participants with diabetes.

	Coefficient	95% Confidence Interval		p-value
**A1c > = 6.5%**	0.42	-0.11	0.95	0.120
**DR Grade**				<0.001
**Mild or no DR**	Reference	.	.	.
**More than mild DR**	-1.24	-1.89	-0.59	<0.001
**Duration of diabetes**	-0.02	-0.04	0.01	0.172
**Age (10-year increase)**	-1.89	-2.26	-1.51	<0.001
**Male**	-0.10	-0.89	0.70	0.813
**Other than White**	-2.07	-2.84	-1.31	<0.001
**Cognitive impairment**	0.13	-0.46	0.73	0.663
**Education score**	0.00	-0.01	0.02	0.702
**BMI**	-0.07	-0.12	-0.02	0.006
**Systolic BP**	0.00	-0.01	0.02	0.677
**HDL**	-0.35	-1.15	0.45	0.397
**Cholesterol lowering medication**	-0.22	-0.95	0.51	0.549
**Metformin**	-0.32	-0.86	0.21	0.238
**Glaucoma**	-4.16	-5.48	-2.83	<0.001
**Cataract surgery**	0.43	-0.82	1.68	0.498
**Spherical equivalent (diopters)**	0.43	0.33	0.52	<0.001
**logMAR visual acuity**	-2.02	-2.66	-1.37	<0.001
**IOP (mmHg)**	-0.04	-0.08	0.01	0.104

A1c = glycated hemoglobin level; BMI = body mass index, HDL = high density lipoprotein, BP = blood pressure, iop = intraocular pressure, RNFL = retinal nerve fiber layer, GC-IPL = ganglion cell inner plexiform layer; DR = diabetic retinopathy.

**Sub-analysis: No diabetes vs diabetes (with no/mild DR).** From 7,687 eyes with diabetes, 5,433 eyes were graded as no/mild and 278 as more than mild. 1,976 eyes were excluded from this analysis because either a) image was missing (n = 94) or b) image was graded as poor quality (n = 1,882).

Average total retinal thickness was lower among those with DM (and no/mild DR) compared to those without DM (4.25 um lower, 95% CI: -4.73, -3.76; p<0.001). The average RNFL thickness was lower among participants with DM (and no/mild DR) compared to those without DM (0.60 um lower, 95% CI: -0.76, -0.44; p<0.001). The average GCL-IPL was thinner among participants with DM and no/mild DR compared to those without DM (1.41 um lower, 95% CI: -1.56, -1.25; p<0.001).

After adjusting for the co-variates mentioned above, the total retinal thicknesses of those with DM (and no/mild DR) was 1.89 um lower compared to those without DM (95% CI: -2.43, -1.34; p<0.001); the average RNFL thickness did not differ among those with DM (and no/mild DR) compared to those without DM (p = 0.417); average GC-IPL thickness was 0.80 microns lower among participants with DM (and no/mild DR) compared to those without DM (95% CI: -0.98, -0.62; p<0.001). S1A–S1C Fig show the differences in average retinal thickness measurements after controlling for co-variates among patients with DM (no/mild DR) and without DM.

After controlling for co-variates, the total retinal thickness decreased 0.39 um (95% CI: -0.62, -0.17; p<0.001) per unit increase in A1c; average RNFL thickness increased 0.21 um (95% CI: 0.14, 0.28; p<0.001) per unit increase in A1c and GC-IPL thickness decreased 0.22 um (95% CI: -0.30, -0.15; p<0.001) per unit increase in A1c.

## Discussion

In this study we sought to address the question regarding the impact of DM on total retinal thickness and thickness of the neuro-retina (mRNFL and GC-IPL), as measured using macular OCT scans, in a large population-based cohort. Our findings show that GC-IPL is thinner among participants with DM compared to non-DM. Additionally, this study shows that thinning persisted after controlling for the many confounding factors associated with retinal thickness measurements, and after excluding participants with DM who had more than mild DR. These findings are consistent with our prior findings from 45 participants with Type 1 DM where we showed progressive GC-IPL thinning over time and those of Aschauer et al showing GC-IPL thinning but no mRNFL thinning over time among patients with Type 2 DM [6, 24]. A recent meta-analysis on this topic also showed a stronger association with GC-IPL thickness compared to mRNFL thickness between DM patients with no DR and controls [25]. Our findings, together with prior work from others, suggest that GC-IPL thickness may be a good marker for evaluation of DRN and animal studies suggest that thinning of GC-IPL may represent loss of ganglion cells [26]. Direct visualization of ganglion cells in-vivo would help further elucidate this.

Our large population-based study also allows us to study ocular and systemic factors associated with GC-IPL thickness among those with and without diabetes. This information is helpful when using GC-IPL clinically as a measure to identify DRN. Factors associated with GC-IPL thickness among patients with DM included age, ethnicity, glaucoma, refractive error, DR grade and logMAR visual acuity. Independent of DR grade, thinner GC-IPL was associated with worse visual acuity, suggesting that DRN, even in the early stages may have an impact on vision. BMI was significant with a small coefficient of -0.07 suggesting that the impact on thickness is likely small. Interestingly gender was not significantly associated with GC-IPL thickness among participants with DM. Khawaja et al reported a lower GC-IPL thickness among men compared to women, and attributed this to the higher rates of glaucoma reported among men [27]. This finding would need to be explored further in additional studies as we know from prior literature that men tend to have higher total retinal thickness measurements compared to women [28]. Consistent with the prospective study by Sohn et al among Type 1 DM patients, and Lim et al [14], A1c of 6.5% or more was not associated with GC-IPL thickness among those with DM [6]. Unlike this study, duration of DM was significantly associated with GC-IPL thickness in the study by Sohn et al. The difference is likely due to the difficulty in measuring duration of disease in patients with Type 2 DM, who may have sub-clinical disease before clinical diagnosis is made. In the study by Sohn et al, all patients had Type 1 DM, and the duration of diabetes was exactly known [6]. In this study, participants, predominantly had type 2 DM, making it difficult to determine the exact date of diagnosis. Aschauer et al, in their study evaluating changes in retinal thickness over time among patients with Type 2 DM, also did not find an association with duration, likely due to the same reason [24]. Further studies are needed to identify patients who are at the highest risk of progressive DRN as treating them with neuroprotective agents, such as brimonidine and somatostatin may be beneficial, as shown in the EUROCONDOR trial [29].

The changes in mRNFL thickness among those with and without DM seemed to be more complex. mRNFL thickness was on average lower in participants with DM compared to non-DM. When we adjusted for confounders, this difference was no longer significant. When we did a sub-analysis only including DM participants with no/mild DR, mRNFL thickness did not differ between those with and without DM. When A1c was used as a predictor, mRNFL thickness increased with increasing A1c levels. There may be several possible explanations for this: 1) neurodegeneration is a process which involves axonal swelling followed by atrophy, our findings suggest that in early stages of DR, mRNFL thickness may be higher than normal; 2) DM affects GC-IPL differentially than mRNFL, mRNFL also includes Muller cells and activated astrocytes/glia, which when activated can lead to thickening of the mRNFL;3) sub-clinical macular edema may also contribute to thicker mRNFL. Baseline data of participants enrolled in the EUROCONDOR trial showed that about a third of patients with Type 2 DM had early microvascular changes but no multifocal ERG changes, suggesting that while some patients have DRN, others do not [30]. Pooling the findings of those with DRN and those without DRN may be minimizing the overall differences in layer thickness between groups. Irrespective of the exact reasons for this finding, our results as well as that of others [24] suggest that GC-IPL thickness may be a more robust marker of early DRN changes compared to mRNFL.

To the best of our knowledge, our study, is one of the largest study to evaluate retinal thickness measures in patients with and without DM, while controlling for multiple confounders, and to report factors associated with GC-IPL thickness among patients with DM.

The strength of our study includes the large numbers, detailed data available on cofounding factors, as well as the rigorous criteria used to define DM. Accuracy of self-reported DM in population-based studies can be limited [31–33]. In order to make our groups as “clean” as possible we also included hemoglobin A1c levels in our determination of DM and excluded patients with pre-diabetes or gestational diabetes.

There are certain limitations to our study, which was retrospective by nature. We were limited by the imaging modalities available in the UKBB dataset. We could not assess peripapillary RNFL thickness and can only comment on the changes in macular RNFL thickness, but as macular centered OCT scans offer the additional advantage of evaluating diabetic macular edema, information regarding thickness measurements from macular scans may be more relevant for clinical practice. Diagnosis of glaucoma was based on self-report and we could have missed some participants with undiagnosed glaucoma or some who incorrectly recalled their diagnosis of glaucoma. However, as the criteria for identifying glaucoma were the same across groups, it is likely that those misclassified, were equally distributed between the two groups. Although, we were limited by one-field fundus photos in our dataset, prior work has shown high agreement between single-field fundus photos and the standard seven field stereoscopic photographs, with a sensitivity and specificity of 78% and 86% respectively, for DR detection [34]. Despite these limitations, our results show, that in a large population-based study GC-IPL thickness is lower among patients with DM compared to those without DM, and this difference persists when considering only those with no/mild DR and after controlling for confounding factors. Our findings also suggest the mRNFL thickness may be more impacted by confounding variables.

Our report adds to a growing body of evidence that DRN is an important component of diabetic eye disease, and that GC-IPL thickness may be a good measure to quantify DRN. Our study further reports factors associated with GC-IPL thickness among those with DM. These include age, ethnicity, refractive error, DR grade, BMI and visual acuity, suggesting that these factors must be considered when evaluating GC-IPL thickness in patients with DM. Additional studies are needed to determine which patients with DM are most likely to develop DRN and to improve our understanding of ganglion cell health by directly visualizing retinal ganglion cells in vivo using newer imaging techniques such as adaptive optics [35]. Our study also showed that that thinner GC-IPL was associated with worse vision independent of DR grade, additional studies are needed to fully evaluate the impact of DRN on functional measures of vision. Finally, as the eye offers unique insights into future risks of disease elsewhere, links between DRN and diabetic peripheral neuropathy and cognitive impairment warrants further study.

## Supporting information

S1 Table a. Patient summary statistics stratified by included vs excluded. b. Eye summary statistics stratified by included vs excluded.
(DOCX)

S2 Table Detailed list of exclusions and reasons for exclusions. (DOCX)

S3 Table Factors association with retinal thickness measurements (Univariate GLMMs). (DOCX)

S1 Fig Difference in retinal thickness measurements between participants with and without diabetes (with no/mild diabetic retinopathy). S1 Fig shows the difference in average thickness measurements (95% confidence interval) between participants with and without diabetes (DM) after adjusting for co-variates and excluding those with more than mild diabetic retinopathy: a) total retinal thickness, b) retinal nerve fiber layer (RNFL) thickness, c) ganglion cell inner plexiform layer (GC-IPL) thickness.
(JPG)
