# Supplementary material for: Detecting retinal neurodegeneration in people with diabetes: Findings from the UK Biobank
Source: PLoS One. 2021 Sep 29;16(9):e0257836. doi: 10.1371/journal.pone.0257836 (PMC8480885; doi:10.1371/journal.pone.0257836)
Supplement: S1 Table — a. Patient summary statistics stratified by included vs excluded. b. Eye summary statistics stratified by included vs excluded. (DOCX) [file pone.0257836.s001.docx]

**Supplementary Table 1a: Patient summary statistics stratified by included vs excluded**

| **Characteristics** | N | Included  (N = 74,422) | N | Excluded  (N = 10,024) | p-value |
| --- | --- | --- | --- | --- | --- |
| **Age(yrs), median (min, max)** | 74,422 | 59.3 (39.2,76.5) | 10,024 | \| 61(40.1,75.5) \| \| --- \| | <0.001 |
| **Education scores, median (min, max)** | 72,920 | 8.9 (0.0,96.2) | 9,831 | 9.2 (0.0,92.5) | 0.052 |
| **BMI, median (min, max)** | 74,072 | 26.6 (12.6,66.0) | 9,993 | 26.7 (13.4,57.7) | 0.013 |
| **HDL, median (min, max)** | 64,003 | 1.4 (0.4,4.1) | 9,050 | 1.4 (0.2,3.5) | <0.001 |
| **Diastolic BP, median (min, max)** | 74,168 | 81 (39.5,142.0) | 10,003 | 81 (49.0,129.0) | 0.242 |
| **Systolic BP, median (min, max)** | 74,167 | 135.5 (65.0,241.0) | 10,003 | 137 (86.5,245.0) | <0.001 |
| **Female, n (%)** | 74,422 | 40,006 (53.8) | 10,024 | 5,344 (53.3) | 0.405 |
| **Ethnicity white, n (%)** | 73,947 | 67,913 (91.8) | 9,944 | 9,204 (92.6) | 0.014 |
| **Incorrect answer on first attempt of prospective memory, n (%)** | 74,013 | 15,650 (21.1) | 9,955 | 2,205 (22.1) | 0.022 |
| **>2 incorrect matches on pairs matching, n (%)** | 74,270 | 47,429 (63.8) | 9,998 | 6,643 (66.4) | <0.001 |
| **Fluid intelligence score < 3, n (%)** | 71,983 | 2,960 (4.1) | 9,660 | 389 (4.0) | 0.722 |
| **Reaction time > 770 milliseconds, n (%)** | 73,598 | 4,127 (5.6) | 9,909 | 627 (6.3) | 0.004 |
| **Cognitive impairment, n (%)** | 73,252 | 14,603 (19.9) | 9,840 | 2,130 (21.6) | <0.001 |
| **BP medication, n (%)** | 74,422 | 8,689 (11.7) | 10,024 | 1,351 (13.5) | <0.001 |
| **Cholesterol lowering medication, n (%)** | 74,422 | 8,686 (11.7) | 10,024 | 1,337 (13.3) | <0.001 |
| p-values for median comparisons using two-sample Wilcoxon rank-sum (Mann-Whitney) test; p-values calculated with exact testing for categorical variables when possible otherwise chi-square test; BMI=body mass index, HDL=high density lipoprotein, BP=blood pressure | | | | | |

**Supplementary Table 1b: Eye summary statistics stratified by included vs excluded**

| **Variable** | N | Included  (N =131,555) | N | Excluded  (N =35,096) | p-value |
| --- | --- | --- | --- | --- | --- |
| **logMAR, median (min, max)** | 131,336 | -0.04 (-1.06,1.35) | 34,691 | 0.02 (-0.46,1.35) | <0.001 |
| **Spherical equivalent, median (min, max)** | 130,214 | 0.2 (-21.1,14.0) | 34,019 | -0.3 (-25.3,14.8) | <0.001 |
| **IOP mmHg, median (min, max)** | 128,008 | 15.4 (5.0,59.8) | 33,668 | 15.7 (0.0,71.3) | <0.001 |
| **Cataract surgery, n. (%)** | 131,550 | 1,806(1.4) | 35,088 | 1,312 (3.7) | <0.001 |
| **Glaucoma, n. (%)** | 131,555 | 1,724 (1.3) | 35,093 | 953 (2.7) | <0.001 |
| p-values estimated using independent GEE; IOP=intra-ocular pressure | | | | | |
