# Supplementary material for: Detecting retinal neurodegeneration in people with diabetes: Findings from the UK Biobank
Source: PLoS One. 2021 Sep 29;16(9):e0257836. doi: 10.1371/journal.pone.0257836 (PMC8480885; doi:10.1371/journal.pone.0257836)
Supplement: S2 Table — (DOCX) [file pone.0257836.s002.docx]

**Supplementary Table 2: Detailed list of exclusions and reasons for exclusions**

**All participants in UKBB with OCT measurements:** 84,446 participants and 166,651 eyes

**Exclusions:** 10,024 participants and 35,096 eyes

**Inclusions:**

|  | Without DM | With DM | Total |
| --- | --- | --- | --- |
| Participants | 69,985 | 4,437 | 74,422 |
| Eyes | 123,868 | 7,687 | 131,555 |

**Details:**

1. Out of 133,922 participants, 84,446 participants had an OCT measurement on at least one eye
2. Exclusions
   1. Gestational DM: n=206 participants
   2. Pre-diabetes: n= 197 participants
   3. Eyes excluded due to poor quality of OCT scan on manual review, surface cost > 62,000, or if undefined region > 0 (n=16,339 right eyes and 15,895 left eyes)
   4. Eyes affected by injury or trauma resulting in loss of vision (if not already excluded): n=682 eyes
   5. Eyes affected by macular degeneration (if not already excluded): n=1,427 eyes
   6. Retinal surgery (if not already excluded): n=524 eyes
   7. IOP < 5 or > 60 (if not already excluded): n = 101 eyes
   8. 1 patient was excluded because they had A1c > 29%
