# Supplementary material for: Detecting retinal neurodegeneration in people with diabetes: Findings from the UK Biobank
Source: PLoS One. 2021 Sep 29;16(9):e0257836. doi: 10.1371/journal.pone.0257836 (PMC8480885; doi:10.1371/journal.pone.0257836)
Supplement: S3 Table — (DOCX) [file pone.0257836.s003.docx]

| **Supplementary Table 3**  **Factors associated with retinal thickness (Univariate GLMMs)** | | | | | | | | |
| --- | --- | --- | --- | --- | --- | --- | --- | --- |
|  | **Total retinal thickness** | | | | **RNFL/GC-IPL thickness** | | | |
|  | **Coefficient** | **95% Confidence Interval** | | **p-value** | **Coefficient** | **95% Confidence Interval** | | **p-value** |
| **Age (10 year increase)** | -2.60 | -2.72 | -2.48 | <0.001 | -0.92 | -1.01 | -0.82 | <0.001 |
| **Education score** | -0.02 | -0.03 | -0.01 | <0.001 | -0.01 | -0.01 | 0.00 | 0.012 |
| **BMI** | -0.18 | -0.20 | -0.16 | <0.001 | -0.04 | -0.06 | -0.03 | <0.001 |
| **HDL** | -0.25 | -0.53 | 0.03 | 0.084 | -0.01 | -0.22 | 0.21 | 0.954 |
| **Diastolic BP** | -0.02 | -0.03 | -0.01 | <0.001 | -0.01 | -0.02 | -0.01 | <0.001 |
| **Systolic BP** | -0.04 | -0.05 | -0.04 | <0.001 | -0.02 | -0.02 | -0.02 | <0.001 |
| **Male** | 3.48 | 3.27 | 3.68 | <0.001 | 0.03 | -0.13 | 0.19 | 0.712 |
| **Other than White** | -6.08 | -6.45 | -5.71 | <0.001 | -0.92 | -1.21 | -0.64 | <0.001 |
| **Cognitive impairment** | -2.08 | -2.34 | -1.82 | <0.001 | -0.55 | -0.75 | -0.35 | <0.001 |
| **Blood pressure medication** | -1.47 | -1.79 | -1.16 | <0.001 | -1.02 | -1.26 | -0.77 | <0.001 |
| **Cholesterol lowering medication** | -1.54 | -1.86 | -1.23 | <0.001 | -1.01 | -1.26 | -0.76 | <0.001 |
| **LogMAR** | -2.04 | -2.24 | -1.84 | <0.001 | -2.19 | -2.59 | -1.78 | <0.001 |
| **Spherical equivalent** | 0.96 | 0.93 | 0.99 | <0.001 | 0.05 | 0.02 | 0.08 | 0.002 |
| **IOP** | -0.06 | -0.07 | -0.05 | <0.001 | -0.05 | -0.07 | -0.03 | <0.001 |
| **Cataract surgery** | 2.17 | 1.67 | 2.68 | <0.001 | -0.69 | -1.36 | -0.01 | 0.048 |
| **Glaucoma** | -6.43 | -6.99 | -5.87 | <0.001 | -3.90 | -4.60 | -3.21 | <0.001 |
| **Diabetes** | -4.57 | -5.00 | -4.14 | <0.001 | -1.19 | -1.52 | -0.85 | <0.001 |
| **A1c (%)** | -2.09 | -2.27 | -1.91 | <0.001 | -0.51 | -0.66 | -0.37 | <0.001 |
| BMI=body mass index, HDL=high density lipoprotein, BP=blood pressure, iop=intraocular pressure, RNFL=retinal nerve fiber layer, GC-IPL=ganglion cell inner plexiform layer; GLMM=generalized linear mixed model | | | | | | | | |
