# Supplementary figures and images for: Detecting retinal neurodegeneration in people with diabetes: Findings from the UK Biobank
Source: PLoS One. 2021 Sep 29;16(9):e0257836. doi: 10.1371/journal.pone.0257836 (PMC8480885; doi:10.1371/journal.pone.0257836)

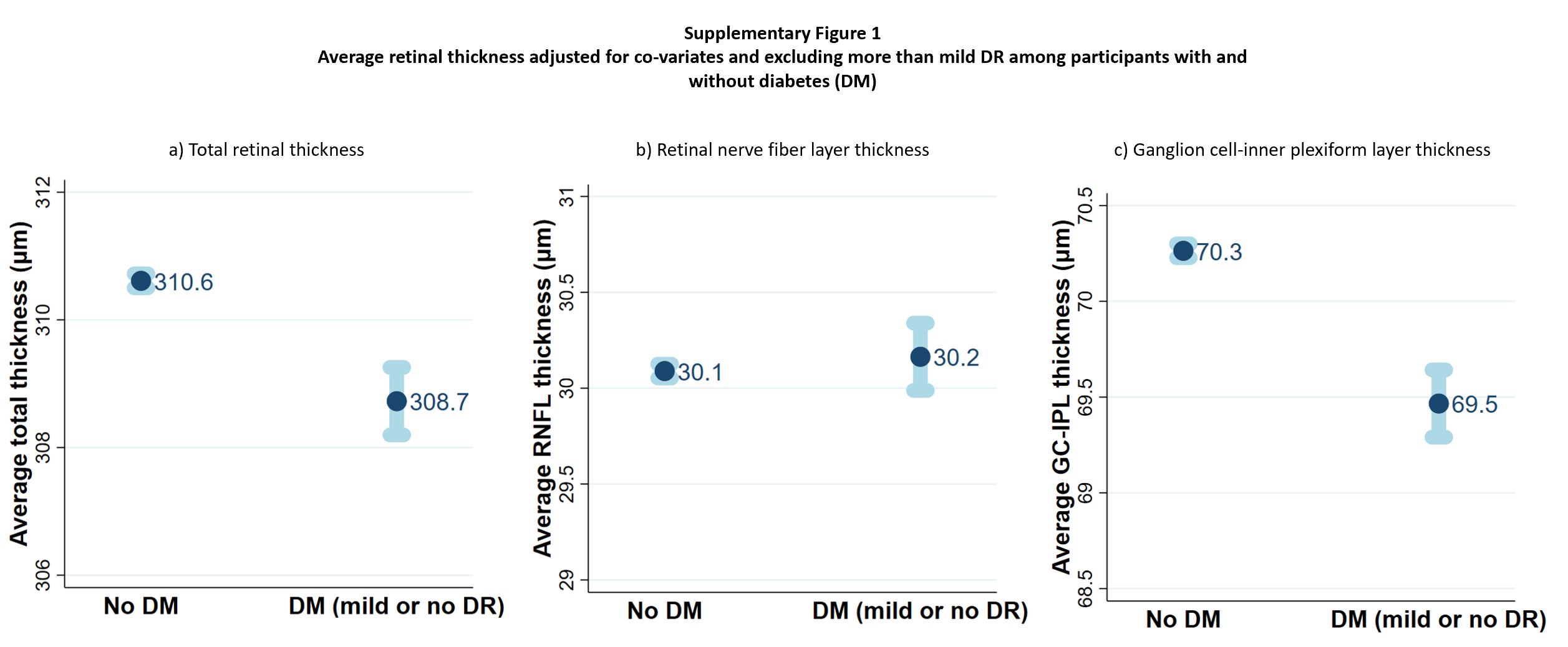

Supplement: S1 Fig — shows the difference in average thickness measurements (95% confidence interval) between participants with and without diabetes (DM) after adjusting for co-variates and excluding those with more than mild diabetic retinopathy: a) total retinal thickness, b) retinal nerve fiber layer (RNFL) thickness, c) ganglion cell inner plexiform layer (GC-IPL) thickness. (JPG) [file pone.0257836.s004.JPG]
